# A Quadruped Micro-Robot Based on Piezoelectric Driving

**DOI:** 10.3390/s18030810

**Published:** 2018-03-07

**Authors:** Qi Su, Qiquan Quan, Jie Deng, Hongpeng Yu

**Affiliations:** School of Mechatronics Engineering, Harbin Institute of Technology, Harbin 150001, China; 18b308002@stu.hit.edu.cn (Q.S.); 16b908017@stu.hit.edu.cn (J.D.); 17s008010@stu.hit.edu.cn (H.Y.)

**Keywords:** legged robot, resonant vibration, miniaturization, bending-bending modes

## Abstract

Inspired by a way of rowing, a new piezoelectric driving quadruped micro-robot operating in bending-bending hybrid vibration modes was proposed and tested in this work. The robot consisted of a steel base, four steel connecting pins and four similar driving legs, and all legs were bonded by four piezoelectric ceramic plates. The driving principle is discussed, which is based on the hybrid of first order vertical bending and first order horizontal bending vibrations. The bending-bending hybrid vibration modes motivated the driving foot to form an elliptical trajectory in space. The vibrations of four legs were used to provide the driving forces for robot motion. The proposed robot was fabricated and tested according to driving principle. The vibration characteristics and elliptical movements of the driving feet were simulated by FEM method. Experimental tests of vibration characteristics and mechanical output abilities were carried out. The tested resonance frequencies and vibration amplitudes agreed well with the FEM calculated results. The size of robot is 36 mm × 98 mm × 14 mm, its weight is only 49.8 g, but its maximum load capacity achieves 200 g. Furthermore, the robot can achieve a maximum speed of 33.45 mm/s.

## 1. Introduction

Micro-robots have shown remarkable application prospects in many fields, such as medical instruments, industrial inspections, and aerospace engineering [1,2,3,4,5]. Much research has been carried out due to the expansive application prospects of micro-robots. According to the different motion forms, micro-robots are mainly distinguished into wheel type, leg type and peristaltic type micro-robot [6]. Meanwhile, micro-robots can be mainly divided into energy field driving type, thermal driving type, pneumatic driving type, and piezoelectric driving type based on different driving principles. Many different types of micro-robots based on different driving principles have been investigated. Lim et al. from Korea Aerospace University developed a very small (70 mm × 9 mm × 9 mm) pneumatic inchworm-like micro-robot in 2008 [7]; the movement of their robot was realized by frequently alternating the inflating and exhausting process, and the movement speed of the robot was changed by changing inflating and exhausting time. However, it was extremely difficult to manufacture and assemble the robot due to the high complexity of its structure. Different from the pneumatic driving type robot, Erdem et al. from University of California, Berkeley utilized the thermal driving principle to manufacture a thermally actuated omnidirectional walking micro-robot in 2010 [8]; the actuation of this robot was achieved by thermal sensitive chips. The basic operating principle was that a thermal sensitive wafer bent to one side of a polymer whose thermal expansion coefficient was small; in this way the single degree of freedom motion was realized. This robot showed the advantages of small size (30 mm × 10 mm × 9 mm), light weight (0.5 g) and high load capacity (3.5 g). However, the limitations of this robot were low speed (only 250 μm/s) and enormous energy consumption. Ishiyama et al. from Tohoku University fabricated a swimming micro-robot in 2001 [9], which was driven by magnetic torque. The movement direction of this robot was controlled by changing the direction of the external magnetic field, and the speed of motion depended on the magnetic field intensity, magnetic field frequency and liquid medium. Although the structure of the robot was very simple, the driving device was very complex, and the moving speed was very low (about 22.5 μm/s).

The driving principles mentioned above have their own merits and drawbacks, and it can be concluded that an excellent micro-robot needs to have the following characteristics: high speed; simple structure, big load capacity and easily controlled [10,11,12]. Piezoelectric driving has the superiority of no electromagnetic radiation, fast response, and simple structure [13,14,15,16,17,18]. Therefore, more and more scholars pay attention to micro-robots that are based on piezoelectric driving. Nowadays, a lot of piezoelectric-walkers have been developed. Yan et al. developed a 3-DOFs mobile robot driven by a piezoelectric actuator [19]. This robot took advantage of a rhombic flexure hinge mechanism and four legs to achieve large stroke 3-DOFs movement. However, this work only focused on the step displacement of the robot, so the load capacity was not well illustrated. Son et al. proposed a precision positioning miniature walking robot based on a piezoelectric unimorph actuator [20]; this robot utilized two standing waves corresponding to the third and fourth bending vibration modes to achieve the driving proposes, and this robot could move forward at a speed of 58.6 mm/s and backwards with velocity of 33.7 mm/s. However, the drawbacks of this robot are quiet obvious, and it only achieve one DOF movement. The power device and circuit design are also very complex, which means that it is difficult to control. Furthermore, this robot shows poor performance on load capacity.

Taking all the merits of micro-robots mentioned above into consideration and inspired by rowing mechanism, a new piezoelectric driving quadruped micro-robot based on the first order vertical and horizontal bending vibrations is proposed in this study. The bending-bending hybrid vibration modes motivate the driving foot to form an elliptical trajectory in space. The structure of the robot is very simple, which only consists of a steel base; four connecting pins and four similar driving legs, and the weight of robot is only 49.8 g.

The basic design of the robot structure and operating principle are shown detail in Section 2. The FEM analysis of the micro-robot is mentioned in Section 3. In Section 4, the Robot is manufactured and its motion is exhibited, and experiments are carried out to reflect output performance of the robot. Finally, Section 5 is the conclusion.

## 2. Structure and Operating Principle

**Figure 1:**
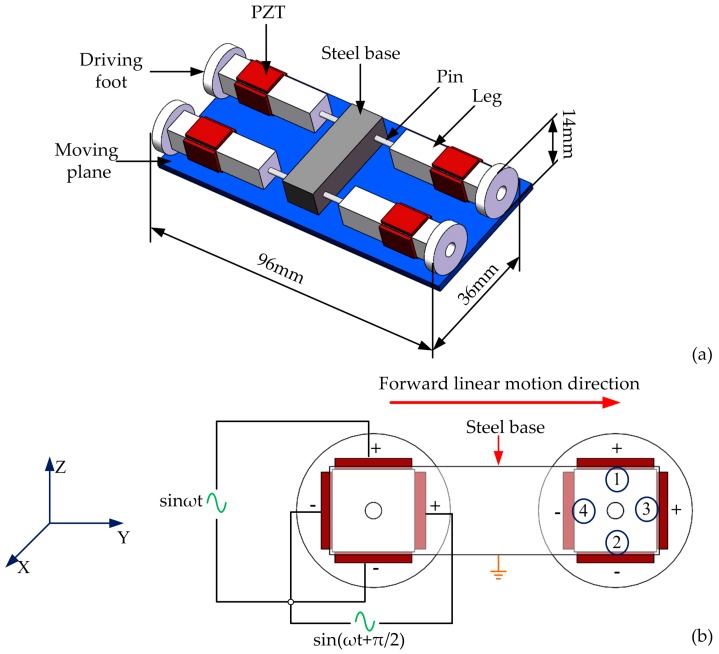
The structure and exciting method design: (**a**) The basic structure of micro-robot; (**b**) The exciting signals.

The basic structure of the proposed micro-robot is shown in Figure 1. The size of the micro-robot is 36 mm × 98 mm × 14 mm, and the robot has one steel base body which connects with four aluminum legs by steel pins. Each leg is bonded by four PZT ceramic plates, the bonding area and position is clearly shown in Figure 1a. It is important to note that the pins are bonded with legs and steel body, which means that the legs cannot rotate. The rigidity and weight of the base are ensured to be big enough by selecting the material and designing the size of the base. Therefore, the vibration will not affect other legs when one leg is vibrating, and each leg is an independent element, which makes it possible to apply different exciting signals to different legs to achieve different movements such as linear motion or turning motion.

The exciting method and arrangements of PZT plates are illustrated in Figure 1b; the piezoelectric ceramic plates are named 1 to 4. It is clearly shown in Figure 1b that PZT-1 and PZT-2 are arranged in opposite directions, as with PZT-3 and PZT-4, and the temporal difference between the two signals applied on the horizontal and vertical piezoelectric ceramic is set as π/2.

**Figure 2:**
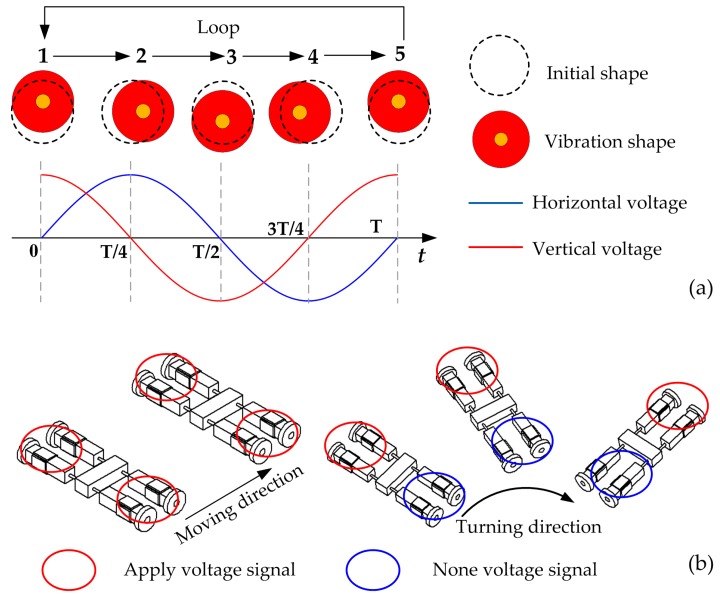
The operating principle: (**a**) Moving sequence of the driving foot; (**b**) Linear motion and turning motion.

The operating principles of linear motion and turning motion are displayed in detail in Figure 2. The moving sequence of one driving foot is presented as 1–2–3–4–5 by applying sine and cosine voltages on the vertical and horizontal bending PZT plates, respectively. The operating sequence can be seen in Figure 2a; it clearly shows that the driving feet will vibrate in elliptical trajectories.

1. In the 0 to *T*/4 interval, the leg bends downwards and forwards at the same time. It reaches the front end at time of *T*/2.
2. The leg bends backwards and downwards when the exciting signal goes into next interval (*T*/4–*T*/2). It reaches the front end at time of *T*/2. In this interval, the driving foot keeps contact with the ground and reaches the lowest position at time of *T*/2.
3. In this step, the leg bends upwards and backwards, and it gradually separates from the ground. The friction force between driving foot and ground gradually decreases, and it completely disappears at time of 3*T*/4.
4. In this step, the leg bends upwards and forwards and reaches the highest position at time of T. In addition, a driving loop has been completed.

When they are vibrating under sequences of 1–2–3–4–5, as mentioned above, each leg is an independent element. Therefore, the four driving legs have the same exciting voltage signals applied if the robot is required to achieve linear motion. The turning motion is roughly the same as the linear motion; the only difference is that the two legs on one side of the robot are in non-voltage states while two legs on the other side also follow the moving sequence. The turning direction is also clearly shown in Figure 2b.

## 3. FEM Analyses 

The resonance frequencies and vibration trajectories of the driving legs are the key factors for the robot motion. This section is devoted to mode and transient analysis. The finite-element method (FEM, ANSYS software) is used to accomplish this process. Modal analysis was used to calculate the resonance frequency of the first vertical bending vibration and horizontal bending vibration, while transient analysis was accomplished to investigate the vibration trajectories of the four feet. The analysis results are illustrated and presented.

### 3.1. Material Selection and Parameters 

The material of steel base is 45# steel with mass density of 7850 $\mathrm{kg}/m^{3}$, Young modulus of 2.10 × $10^{11}$ N/$m^{2}$ and Poisson ratio of 0.269, and the material of legs and driving feet are 7075 aluminum alloy with mass density of 2810 $\mathrm{kg}/m^{3}$, Young modulus of 7.17 × $10^{10}$ N/$m^{2}$ and Poisson ratio of 0.33. The PZT ceramics of the actuator are PZT-4 with density of 7600 kg/m^3^, whose physical parameters are as follows.

(1) $$d=\left[\begin{array}{cccccc} 0 & 0 & 0 & 0 & 5 & 0\\ 0 & 0 & 0 & 5 & 0 & 0\\ -1.6 & -1.6 & 3.3 & 0 & 0 & 0 \end{array}\right]\times 10^{-10}\;C/N$$

(2) $$c^{E}=\left[\begin{array}{cccccc} 14.3 & 7.85 & 7.85 & 0 & 0 & 0\\ 7.85 & 14.3 & 7.85 & 0 & 0 & 0\\ 7.85 & 7.85 & 11.5 & 0 & 0 & 0\\ 0 & 0 & 0 & 2.6 & 0 & 0\\ 0 & 0 & 0 & 0 & 2.45 & 0\\ 0 & 0 & 0 & 0 & 0 & 2.45 \end{array}\right]\times 10^{10}\;{N/m}^{2}$$

(3) $$\varepsilon^{T}=\left[\begin{array}{ccc} 8.1 & 0 & 0\\ 0 & 8.1 & 0\\ 0 & 0 & 6.7 \end{array}\right]\times 10^{-9}\;F/m$$

where *d*, *c^E^* and *ε^T^* are the piezoelectric matrix, the stiffness matrix, and the dielectric matrix, respectively.

### 3.2. Modal and Transient Analysis

**Figure 3:**
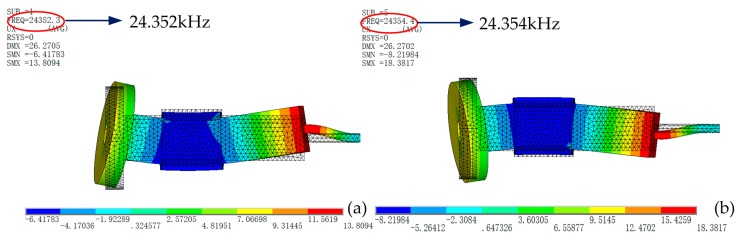
Vibration modes by modal analysis: (**a**) Vertical bending mode; (**b**) Horizontal bending mode.

**Figure 4:**
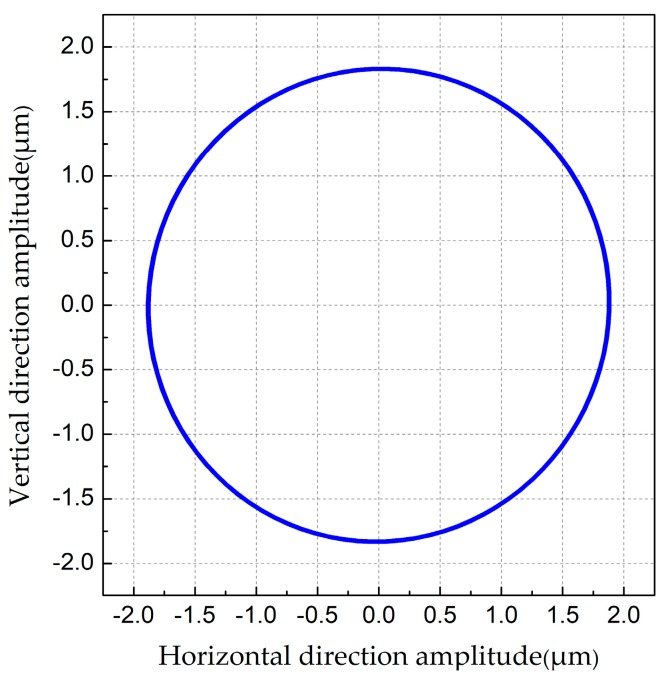
The movement trajectory of driving foot in one period.

The FEM model of one leg was built by ANSYS software, modal analysis was accomplished by applying fixed boundary on the end of the connecting pin. The vibration modes shown in Figure 3 illustrates that the resonance frequencies of vertical and horizontal bending vibrations are 24.352 kHz and 24.534 kHz respectively. The vibration amplitude of the driving feet will significantly influence the driving results. Therefore, it is important to verify whether the amplitude is strong enough. The transient response was used to calculate the vibration amplitude of the driving foot under resonance frequency (24.353 kHz). Meanwhile, the amplitude of applied voltage is 200 V_p-p_. It can acquire the movement trajectory at any point of driving foot under such voltage and frequency conditions. The movement trajectory of one selected point is shown in Figure 4 when the steady state is reached. The horizontal and vertical amplitudes of one selected driving point are about 1.8 μm.

## 4. Experiment

**Figure 5:**
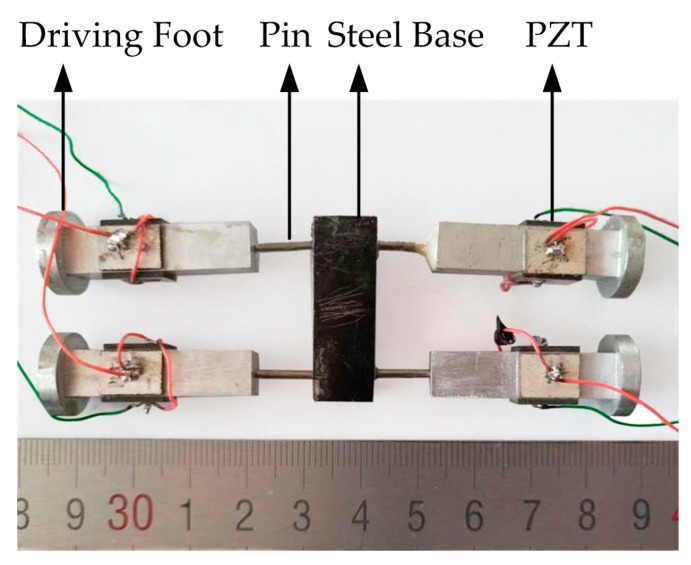
Prototype of the proposed robot.

The prototype of proposed robot was fabricated under the parameters shown in Figure 1. The body and feet of the robot were fabricated by high-precision CNC machining center to ensure manufacturing precision. In addition, the steel base serves as preload and a platform to bear weight. The aluminum leg and steal pin are fixed by adhesion. Both sides of the PZT plates have silver electrodes and the PZT plates are boned on the leg by conductive adhesion. In addition, two different color wires are used to excite the robot; the red wires are used to excite the vertical PZTs while the green wires are used to excite the horizontal PZTs. The prototype of the robot is shown in Figure 5. The weight of the robot is 49.8 g, and its size is 36 mm × 96 mm × 14 mm.

### 4.1. Vibration Analysis of Laser Measurement 

**Figure 6:**
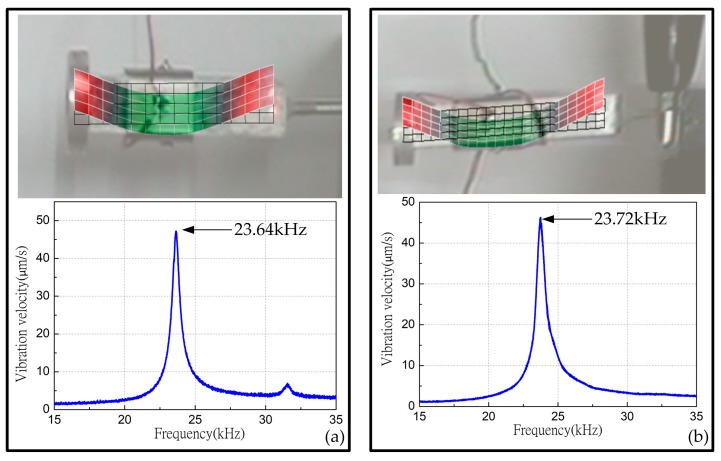
The vibration test results of one leg: (**a**) Real vibration shape and the corresponding frequency in horizontal direction; (**b**) Real vibration shape and the corresponding frequency in vertical direction.

The vibration modes of one leg of the robot were measured by using a scanning laser Doppler vibrometer (PSV-400-M2, Polytec, Waldbronn, Germany) to gain the real vibration shapes and the corresponding frequencies. During the measurement, the two orthogonal surfaces of the driving leg were selected as the test areas for the measurements of the horizontal and the vertical modes. From the vibration shape and the vibration velocity response spectrums shown in Figure 6, it can be clearly seen that the first vertical and horizontal bending resonance frequency is tested to be about 23.64 kHz and 23.72 kHz, respectively. The first vertical and horizontal bending resonance frequency of other three legs are 23.76 kHz; 23.80 kHz; 23.64 kHz; 23.69 kHz; 23.54 kHz; 23.61 kHz, respectively. It is obvious that the two resonance frequencies are very close, so it is certain that we can use the same working frequency to excite the horizontal and vertical vibrations effectively.

The test results of the driving foot agree well with the vibration shapes shown in Figure 3, but the tested resonance frequencies of the horizontal and vertical modes show a decrease of 713 Hz and 634 Hz, respectively, compared with the FEM calculates one. The main reasons caused the discrepancies are the machining and assembling errors, as well as that the material parameters used in the FEM analysis are not consistent with the actual ones. The parameters we used in the FEM model are the standard parameters provided by the manufacturer, but there are individual differences in the elements in the actual manufacturing process.

### 4.2. Experiment of Linear Motion and Turning Motion

**Figure 7:**
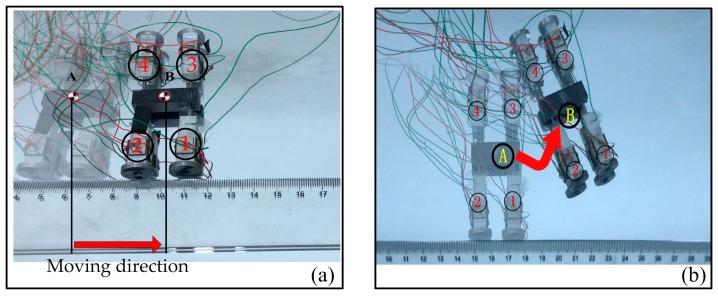
Movement test of the robot: (**a**) Linear motion; (**b**) Turning motion.

The experimental device and setup is very simple, which only needs one ultrasonic power supply. All tests are carried out on the smooth glass plane, the proposed robot cannot move on a surface with very large roughness due to the vibration amplitudes of the driving feet being about 2 µm. Each foot is numbered, which is shown in Figure 7a,b, for detailed description. The linear and turning motion tests were carried out as follows. For linear motion of the robot, the robot the same voltage signals were applied to the four driving feet and they were vibrating synchronously. The robot moved straight on the glass surface with the exciting voltage of 150 V_p-p_ and frequency of 23.64 kHz. Its velocity was about 25 mm/s. For the turning motion, the same voltage signals were only applied on foot 1 and 2, and foot 3 and 4 were kept in non-voltage states. The robot turned right because of the weight and friction forces of foot 3 and 4, as shown in Figure 6b. However, the friction forces were not big enough due to the moving test being carried out on the smooth glass surface, so the slip phenomenon appeared on foot 3 and 4. As a result, the trajectory of turning motion was actually an ellipse. If we keep foot 1 and 2 in non-voltage states and excite foot 3 and 4, the robot will turn left.

### 4.3. Output Performance of the Robot

**Figure 8:**
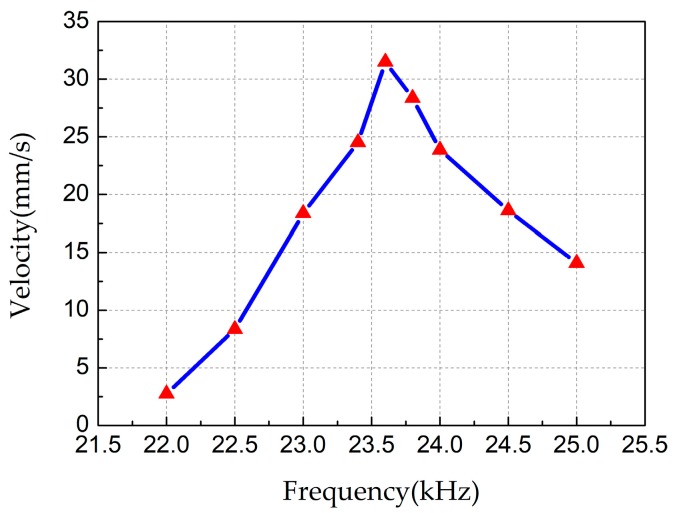
Plot of the output velocity versus different input frequencies.

The output performance test of the robot mainly consists of the following three aspects: velocity versus frequency, velocity versus voltage and velocity versus preload. All tests were studied on a smooth glass. First, the test of the relationship between frequency and response velocity was carried out, as shown in Figure 8, during which voltages of 200 V_p-p_ were applied and no load was carried. The moving speed increases first and then decreases with the increase of frequency and reaches the maximum speed of 31.48 mm/s at 23.6 kHz. Obviously, the resonant frequency of the robot is about 23.6 kHz.

**Figure 9:**
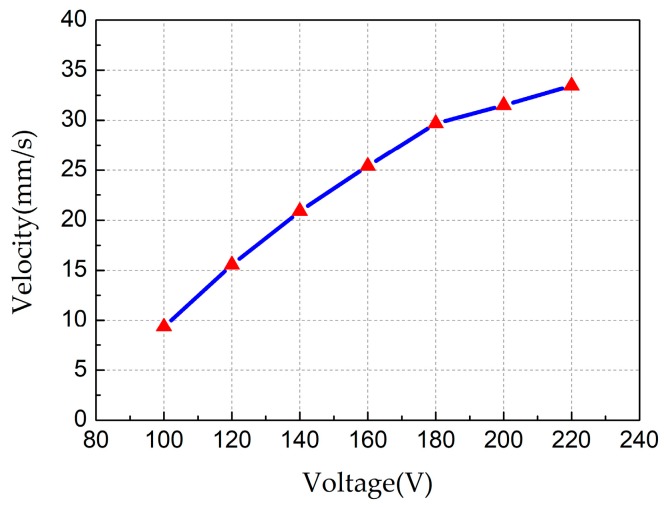
Plot of the output velocity versus the different input voltages.

Then, the performance of the output velocity versus the different input voltages was measured, as shown in Figure 9, in which the working frequency was 23.6 kHz and no load was carried. We can see that the speed of the robot increases as the voltage increases, and the maximum speed is 33.45 mm/s at voltage of 220 V_p-p_.

**Figure 10:**
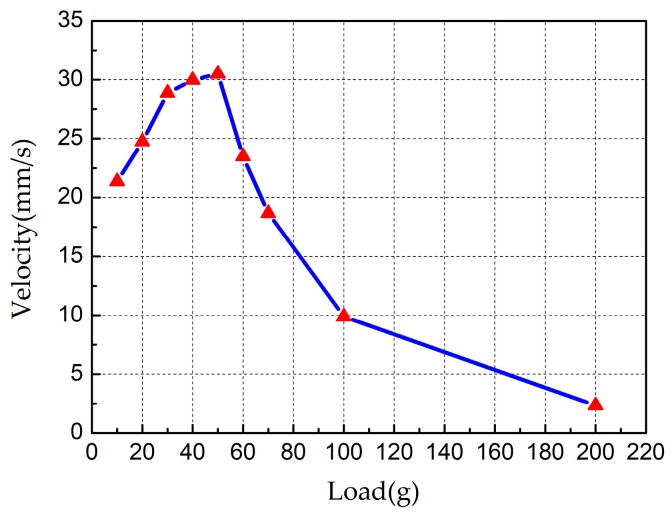
Plot of the output velocity versus different loads.

Finally, the load-carrying capacity of the robot was tested. The excitation voltage was set as 150 V_p-p_ and frequency of 23.6 kHz; the results are shown in Figure 10. The speed increases slowly and then decreases rapidly with the increase of load. The maximum speed is 30.5 mm/s at load of 50 g. Meanwhile, it can be seen that the optimal load of the robot is about 50 g, when the load is larger than optimal load, the robot speed decreases rapidly. Besides, the maximum load capacity of the proposed robot is 200 g and its speed is about 2.35 mm/s.

Some micro-robots are summarized in Table 1, where a comparison is given in terms of their actuation mechanism, DOF of motion, speed, size, weight, and load capacity. It shows the superiority of the proposed robot. The proposed robot can achieve 3-DOF movements and has better load capacity, in comparison with a pneumatic robot [7]. This robot also shows advantage of faster speed and bigger load capacity when it compares to the thermal robot [8] and magnetic robot [9]. The proposed robot shows merits in weight, speed, and load capacity when it compares with Yan’s robot [19]. Finally, compared with Son’s robot [20], this robot has characteristics of multiple degrees of freedom motion and better load capacity. Therefore, the proposed quadruped piezoelectric driving micro-robot achieves high speed and big load capacity with a small weight.

## 5. Conclusions

A new quadruped piezoelectric driving micro-robot based on bending-bending resonant vibration modes was proposed. Sinusoidal voltage signals with phase difference of 90 degrees were applied on the piezoelectric ceramics to obtain the circular trajectory movement of the driving foot. Operating principles were planned, discussed, and simulated by the FEM (ANSYS software 10.0). A prototype of the robot was fabricated, and its experimental system was established. Linear and rotary movement tests were carried out. The output characteristics of the prototype were tested; a maximum speed of 33.45 mm/s and a maximum load capacity about 200 g were obtained. This paper provides a new research vision for micro-robots with high speed and big load capacity by using bending-bending hybrid modes. Future works should mainly focus on the following aspects: design and fabrication of a new type of signal driving control system, developing the positioning ability under a closed-loop control and realizing the micron or even nanometer stroke of the micro-robot.

## Figures and Tables

**Table 1:** The comparison of mentioned robots.

Authors	Actuation Mechanism	Size	Weight	DOF	Speed	Load Capacity
Lim et al. [7]	Pneumatic	70 mm × 10 mm × 10 mm	Not specified	1	50 mm/s	Not specified
Erdem et al. [8]	Thermal	30 mm × 10 mm × 0.9 mm	0.478 g	3	Linear:0.25 mm/s. Rotational: 0.33 deg/s	3.5 g
Ishiyama et al. [9]	Magnetic	12 mm × 2 mm × 2 mm	Not specified	Not specified	22.5 μm/s	Not specified
Yan et al. [19]	Piezoelectric	55 mm × 35 mm × 20 mm	65 g	3	0.24 mm/s	Not specified
Son et al. [20]	Piezoelectric	55 mm × 45 mm × 19 mm	23.25 g	1	58.6 mm/s	Not specified
This work	Piezoelectric	36 mm × 98 mm × 14 mm	49.8 g	3	33.45 mm/s	200 g
